# miRNA-mRNA crosstalk in myocardial ischemia induced by calcified aortic valve stenosis

**DOI:** 10.18632/aging.101751

**Published:** 2019-01-16

**Authors:** Chenyang Duan, Zhezhe Cao, Fuqin Tang, Zhao Jian, Chunshui Liang, Hong Liu, Yingbin Xiao, Liangming Liu, Ruiyan Ma

**Affiliations:** 1 State Key Laboratory of Trauma, Burns and Combined Injury, Second Department of Research Institute of Surgery, Daping Hospital, Army Medical University, Chongqing 400042, P. R. China; 2 Department of Cardiovascular Surgery, Xinqiao Hospital, Army Medical University, Chongqing 400037, P. R. China

**Keywords:** calcified aortic valve stenosis (CAVS), myocardial ischemia, mitochondrial dysfunction, hub genes, miRNA-mRNA crosstalk

## Abstract

Aortic valve stenosis is the most common cause of morbidity and mortality in valvular heart disease in aged people. Both microRNA (miRNA) and mRNA are potential targets for the diagnosis and therapeutic intervention of myocardial ischemia induced by calcified aortic valve stenosis (CAVS), with unclear mechanisms. Here, 3 gene expression profiles of 47 male participants were applied to generate shared differentially expressed genes (DEGs) with significant major biological functions. Moreover, 20 hub genes were generated by a Weighted Genes Co-Expression Network Analysis (WGCNA) and were cross-linked to miRNA based on miRanda/miRwalk2 databases. Integrated miRNA/mRNA analysis identified several novel miRNAs and targeted genes as diagnostic/prognostic biomarkers or therapeutic targets in CAVS patients. In addition, the clinical data suggested that myocardial hypertrophy and myocardial ischemia in CAVS patients are likely associated with hub genes and the upstream regulatory miRNAs. Together, our data provide evidence that miRNAs and their targeted genes play an important role in the pathogenesis of myocardial hypertrophy and ischemia in patients with CAVS.

## Introduction

Myocardial ischemia (MI) is a pathological condition in which the cardiac blood perfusion as well as oxygen supply reduce significantly. Many factors may induce myocardial ischemia, such as valve disease, changes in blood viscosity, and myocardial lesions, etc. During aging, calcified aortic valve disease (CAVD) gradually increases with enhanced myocardial hypertrophy, myocardial ischemia, and cardiac dysfunction [1,2], eventually leading to severe aortic stenosis (AS), also known as calcified aortic stenosis (CAVS) [3]. The current effective treatment for CAVS is valve replacement but the outcome of medical trials of therapies aiming to delay or prevent the progression of myocardial ischemia induced by CAVS are not satisfactory [4,5]. The Current preconditioning and post-conditioning measures for myocardial ischemia are mainly aimed at calcium overload, increased ROS and inflammatory reactions after CAVS, which are closely associated with cardiac mitochondria functions. Therefore, we hypothesis that the prognosis of myocardial ischemia induced by CAVS may be related to altered mitochondrial function in CAVS and improvement of mitochondria functions may be useful to assess or delay the progression of myocardial ischemia after CAVS.

Valvular calcification and myocardial fibrosis precede the development of aortic stenosis and thereby myocardial hypertrophy as well as myocardial ischemia [6]. It was reported that genetic factors play important roles in valvular calcification [7], but the key gene mutations in valvular calcification is still unclear.

Simultaneously, small non-coding RNAs, also known as microRNAs (miRNAs), may also influence the development of aortic stenosis. The study of Beaumont J et al. showed that the down-regulation of miRNA-19b may increase myocardial stiffness [8]. The study of Li J et al. showed that the up-regulation of miRNA-9 may improve high glucose-induced cardiac fibrosis [9], and the down-regulation of miRNA-141 may induce the calcification of aortic stenosis [10]. However, the association between genetic variation and miRNAs in the development of CAVS is limitedly known.

In this study, we used systematic bioinformatics approaches to determine the potential diagnostic/ prognostic and therapeutic targets of CAVS and validated our findings on clinical samples. We also focused on the myocardial ischemia process after CAVS and determine its relationships with cardiac mitochondrial functions. A better understanding of the genetic variability of valve calcification and its potential mechanisms on organelle levels may contribute to elucidate the pathogenesis of valvular heart disease, like CAVS, and promote the development of new therapies in ischemic cardiomyopathy.

## RESULTS

### Differentially expressed mRNA profiles in calcified aortic valve tissue (CAVS)

**Figure 1:**
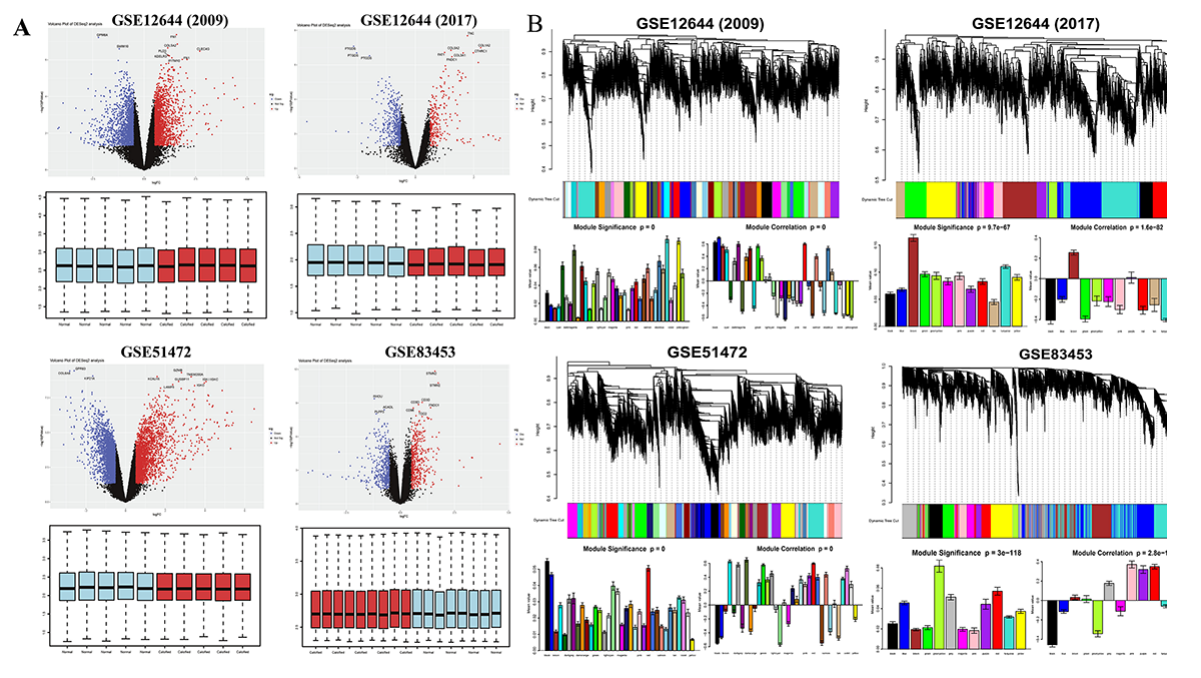
**Overview of DEGs between calcified aortic valve stenosis and normal aortic valve tissues.** (**A**) Volcano plot of DEGs and boxplot of normalized gene expression profiles. In the volcano plots, each color dot represents a downregulated or upregulated gene; The red color indicated high expressed genes and the blue color indicated low expressed genes, above and below the median, respectively. (**B**) Gene modules’ dendrogram plots of DEGs, and bar plots of eigengenes’ module significance and correlation. In the dendrogram plots, each leaf (short vertical lines) in the dendrogram corresponds to a gene and the branches are expression modules of highly interconnected groups of genes with a color to indicate its module assignment.

DEGs were identified separately in each paired group using identical analysis package and threshold (p-value < 0.05, logFC > 0.5) after normalization (boxplots of each normalized gene expression profile are shown in Figure 1A). The analysis yielded 9125 DEGs in GSE12644 (2009), 3137 DEGs in GSE12544 (2017), 13575 DEGs in GSE51472 and 5425 DEGs in GSE83453 (volcano plots of each data profile are shown in Figure 1A, Table 1).

**Table 1:** Characteristics of the gene expression profiles.

GEO ID	Contributors	Country	Year	Sample size	Mean age	Platform	Probes	DEGs
GSE83453	Bossé Y	Canada	2016	male: 17	62.4 ± 4.7	Illumina HumanHT-12 V4.0	47323	5425
GSE51472	Rysä J	Finland	2015	male: 10	NA	HG-U133_Plus_2	54675	13575
GSE12644	Bosse Y, Pibarot P, Mathieu P	Canada	2009	male: 10	65.4 ± 5.8	HG-U133_Plus_2	54675	9125
			2017	male: 10	55.7 ± 10.8			3137

DEGs: differentially expressed genes (P < 0.05); NA: not available.

Next, significant gene modules were generated with WGCNA for enrichment analysis to study their association with CAVS. The dynamic branching method was used to identify gene modules for each set of DEGs and to define them as branches of the resulting cluster tree in each individual dendrogram (the gene dendrogram plots are shown in Figure 1B). In addition, genes within a given module were summarized with the module eigengenes (bar plots of module significance are shown in Figure 1B); these plots were considered the best summary of standardized module expression data.

**Figure 2:**
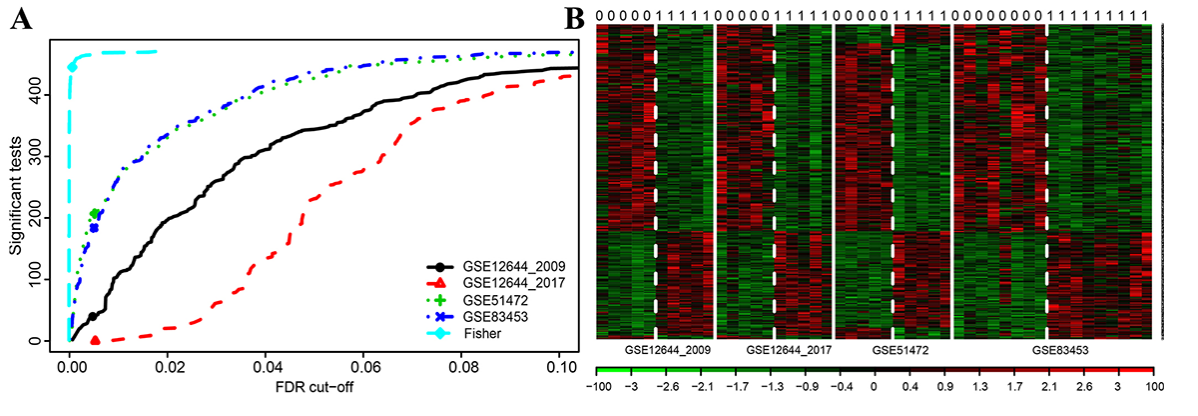
**Microarray analysis for DEGs of integrated multiple datasets.** (**A**) Fisher’s method tests the integrated multiple profiles of the shared DEGs’ expression; (**B**) Integrated heat map of significantly expressed DEGs with each row representing a probe and each column representing a sample. Expression levels are depicted according to the color scale, shown at the bottom. The red color indicated high expressed genes and the blue color indicated low expressed genes, above and below the median, respectively. The magnitude of deviation from the median is represented by the color saturation.

Finally, 471 shared DEGs were filtered out of data profiles and subjected to a microarray analysis. Fisher’s exact test was applied to identify DEGs by integrating multiple datasets with a combined significance test line corresponding to a maximum FDR cut-off value of 0.1 (Figure 2A). An integrated heatmap with an FDR cut-off value (FDR = 0.05) of the shared DEGs was also generated by microarray analysis (Figure 2B).

### Functional enrichment related to mitochondria

**Figure 3:**
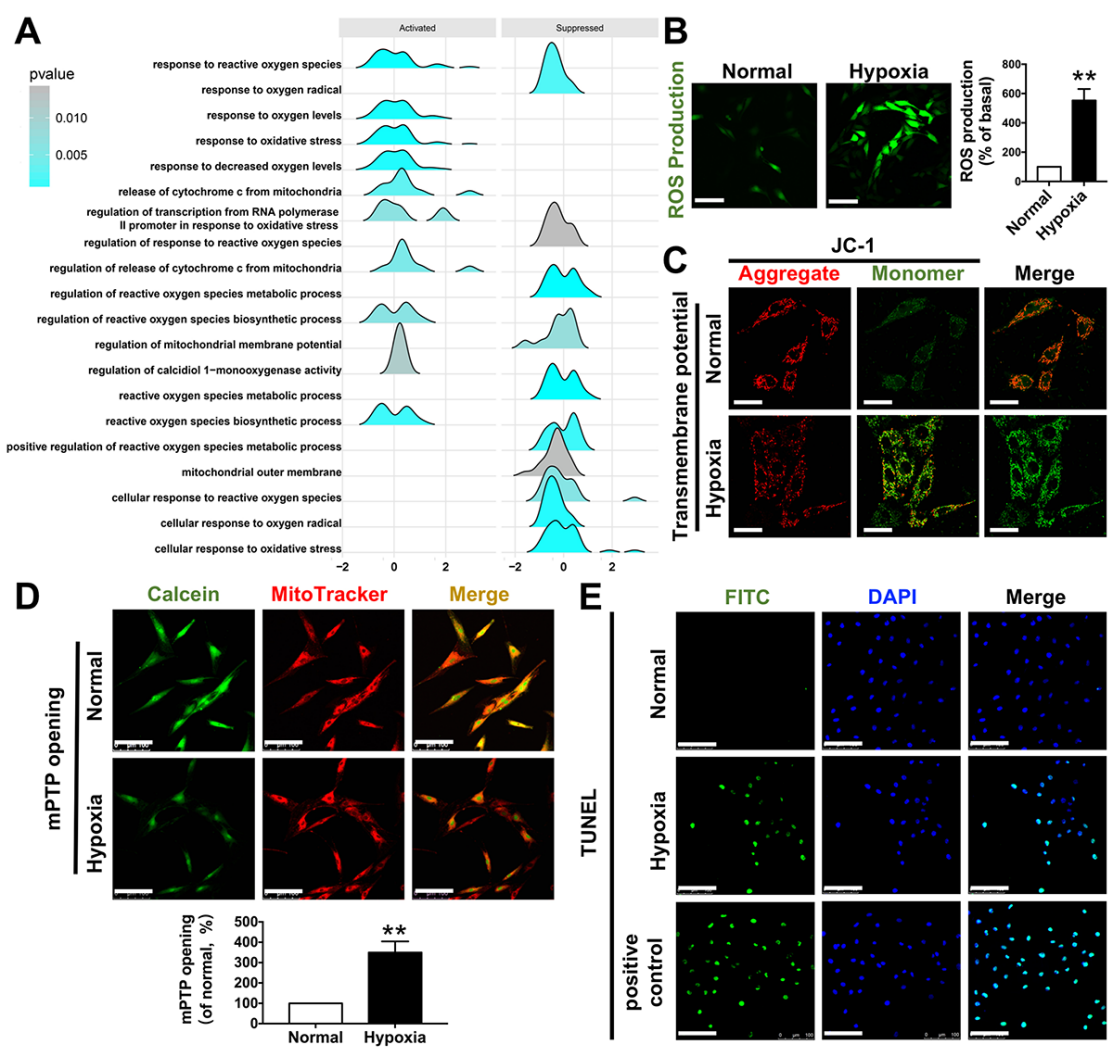
**Functional enrichment related to mitochondria.** (**A**) GSEA enrichment results related to mitochondrial functions; (**B**) ROS production after H9C2 hypoxic simulated myocardial ischemia, bar=100μm; (**C**) mitochondrial transmembrane potential after H9C2 hypoxic simulated myocardial ischemia, bar=50μm; (**D**) mPTP opening after H9C2 hypoxic simulated myocardial ischemia, bar=100μm; (**E**) TUNEL staining after H9C2 hypoxic simulated myocardial ischemia, bar=100μm. *P<0.05, **P<0.01.

Gene annotation and functional enrichment analysis of these shared DEGs were performed using the ClusterProfiler software package in R to determine important biological pathways related to mitochondria. As shown in Figure 3A, the response to reactive oxygen species (ROS) was elevated while the mitochondrial membrane potential was suppressed, suggesting occurrence of mitochondrial dysfunctions during myocardial ischemia induced by CAVS. The exact pathogenesis of CAVS is complicated and could not be mimicked in an in vitro experiment perfectly, since a variety of diverse factors are found to induce development of CAVS. However, the eventual damage to the myocytes in CAVS at least partially (if not predominantly) results from hypoxia. Therefore, hypoxia exposure can be rather informative for studying molecular mechanism. Hence, we carried out hypoxia treatment on H9C2 cells. We found that the ROS production in H9C2 cells increased 4 times after hypoxia (Figure 3B). As for mitochondrial transmembrane potential (ΔΨm), JC-1 monomer, which represents the depolarization of ΔΨm after hypoxia, was significantly increased (Figure 3C). Moreover, the functional enrichment analysis showed that the release of cytochrome C from mitochondria was increased, possibly due to mPTP opening and apoptosis in CAVS (Figure 3D-E).

**Figure 4:**
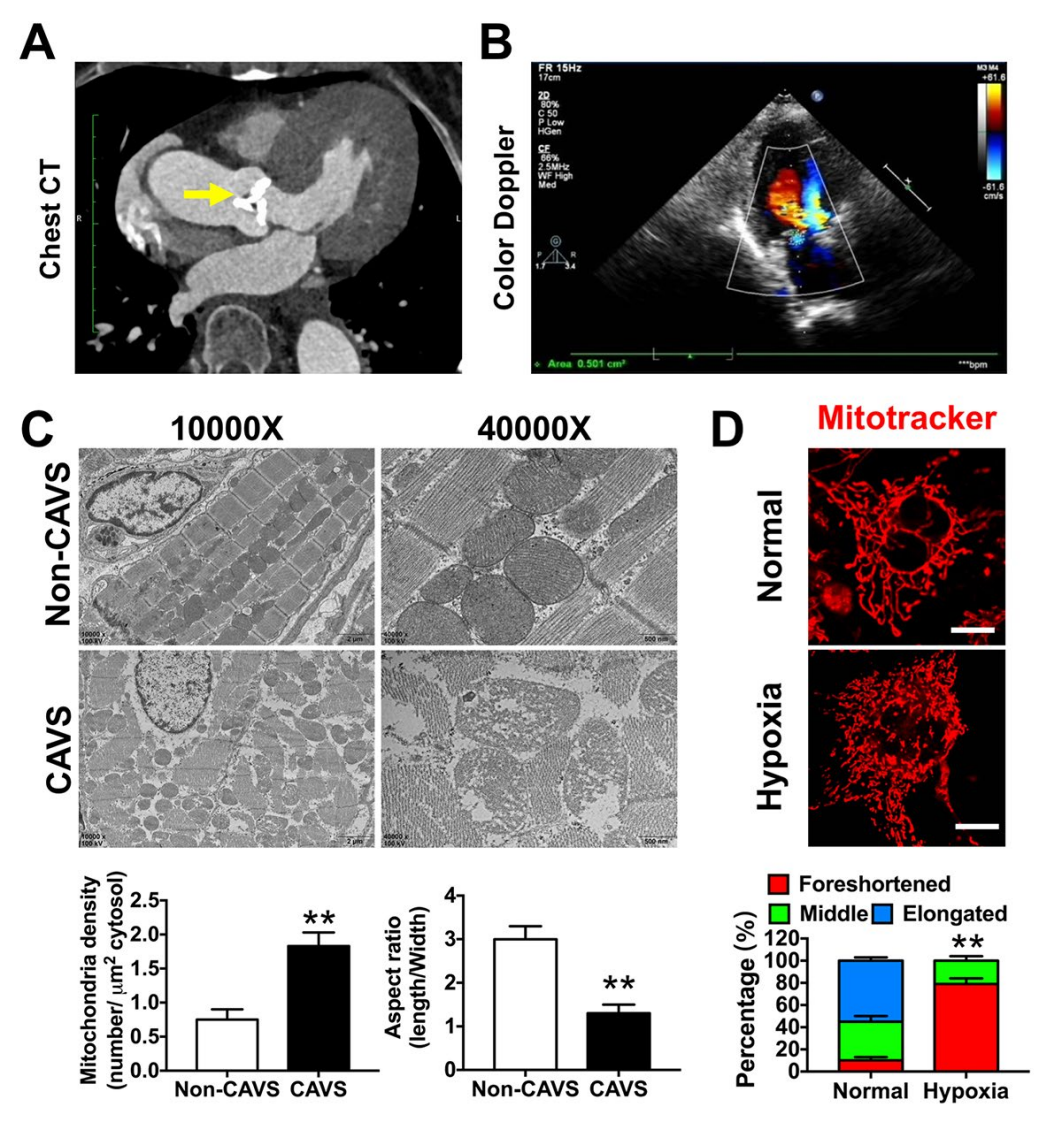
**Mitochondrial dysfunctions after myocardial ischemia in CAVS patients.** (**A**) The chest CT of the CAVS patient. The yellow arrow refers to the calcified aortic valve; (**B**) The cardiac multifunctional color Doppler ultrasound image of the CAVS patient; (**C**) The electronic microscopy observation of mitochondria in CAVS myocardial tissues; (**D**) Confocal microscopy observation of mitochondrial morphology in H9C2 hypoxic simulated myocardial ischemia, bar=10μm. Quantitation is done in triplicate and scored into three categories: foreshortened, middle and elongated mitochondria. *P<0.05, **P<0.01.

Next, we studied mitochondrial variation in CAVS patients. The chest CT showed a classical calcified aortic valve, which caused cardiac hypertrophy (Figure 4A). The cardiac multifunctional color Doppler ultrasound image of the patient showed serious valve stenosis (the opening area of aortic valve is 0.5 cm^2^) and myocardial ischemia in CAVS (Figure 4B). After valve replacement, the calcified aortic valve was taken for electronic microscopy observation, showing that mitochondrial density was significantly increased and the aspect ratio was significantly decreased in CAVS group (*p*<0.05) (Figure 4C). The mitochondrial crista during high magnification observation in CAVS group was severely destroyed, suggesting that CAVS-induced myocardial ischemia may cause mitochondrial dysfunction. Mitochondria morphology in H9C2 cells labeled with Mitotracker in hypoxia showed that mitochondrial fragments significantly increased (*p*<0.05) (Figure 4D), supporting the hypothesis of the existence of mitochondrial dysfunction in CAVS-induced myocardial ischemia *in vitro*.

### Gene co-expression analysis reveals hub genes

**Figure 5:**
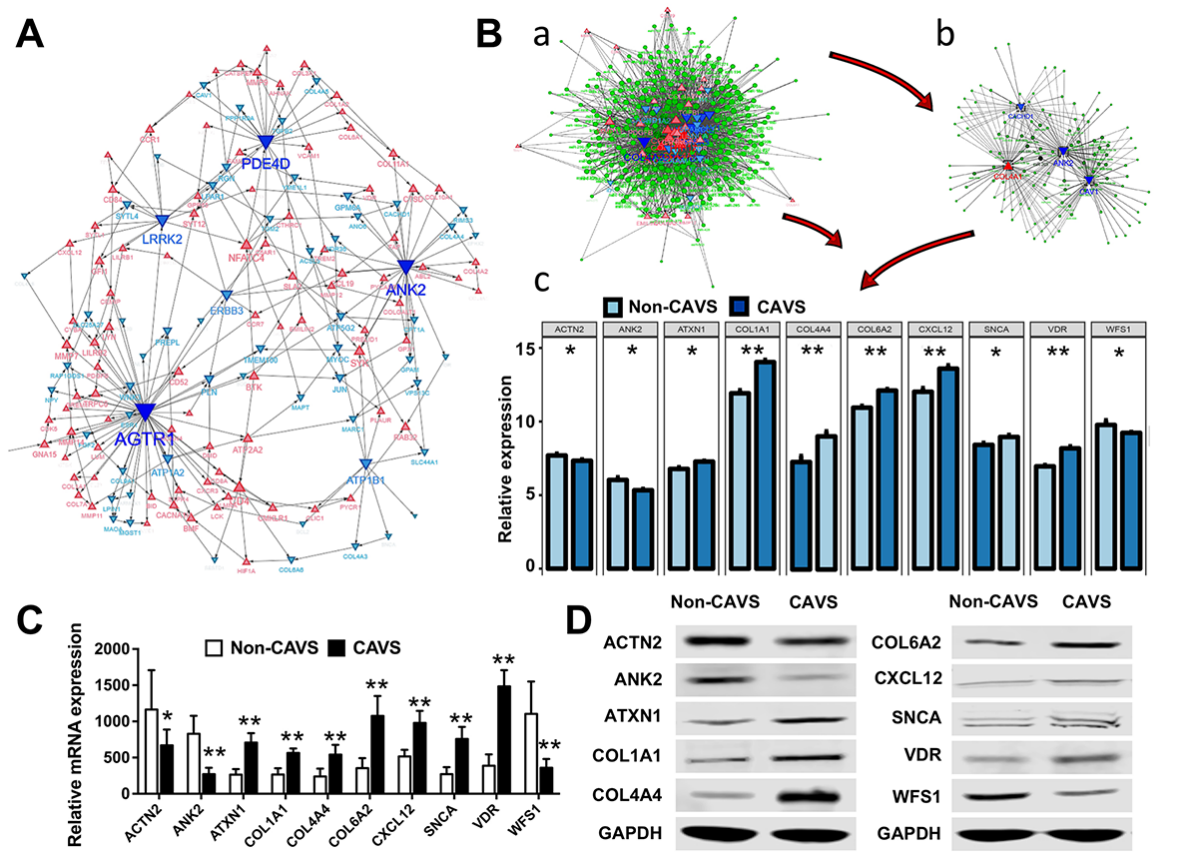
**Biological processes and hub genes related to mitochondrial functions in CAVS.** (**A**) Co-expression network approaches to reveal hub genes associated with mitochondrial functions in CAVS; (**B**) a. overview of hub genes and connected miRNAs. b. subnetwork of the integrated miRNAs and hub genes. The triangle and dot represent the hub genes and miRNA, respectively. Red color and up-direction represent up-regulated hub genes, blue color and down-direction represent down-regulated hub genes. The size color saturation of the nodes was weighted by the connectedness of the hub gene interacted with other miRNAs. c. two tail T-test of top-ten hub genes expression based on data profiles; C. Real-time PCR results of mRNA expression of these top-ten hub genes based on clinical samples; D. Western Blot results of protein expression of these top-ten hub genes based on clinical samples; *P<0.05, **P<0.01.

Hub genes play key roles in regulating biological processes. Hub genes are defined on the basis of the relevance of each gene to the corresponding module eigengene, which defines robust biomarkers [11] or refers to the sum of the proximities to the module genes. Based on a threshold of 0.35 and a connected degree of more than 5 in the entire co-expression network of shared DEGs, 20 potential hub genes were found possibly associated with the functional enrichment related to mitochondria (Figure 5A). The 20 potential hub genes include: ANK2, VDR, SNCA, CXCL12, COL6A2, COL4A4, COL1A1, WFS1, ATXN1, ACTN2, ATP1A2, CACHD1, TGFBI, CAV1, PDGFB, COL3A1, COL4A1, COL4A2, COL4A3, and WNK3 (Table 2). The diagnostic characteristics of these twenty hub genes are shown in Supplementary Figure 1.

**Table 2:** Hub genes and cross-linked miRNAs.

**mRNA**	**Counts**	**Cross-linked miRNAs**
**CXCL12**	76	miR-590-3p, miR-203, miR-410, miR-186, miR-539, miR-340, miR-106a, miR-17, miR-20a, miR-20b, miR-93, miR-106b, miR-23a, miR-23b, miR-29b, miR-519d, miR-9, miR-130a, miR-29a, miR-29c, miR-301a, miR-301b, miR-454, miR-495, miR-144, miR-200b, miR-381, miR-429, miR-543, miR-130b, miR-302c, miR-302d, miR-372, miR-373, miR-494, miR-182, miR-300, miR-302a, miR-302b, miR-302e, miR-520a-3p, miR-520b, miR-520c-3p, miR-520d-3p, miR-520e, miR-135a, miR-135b, miR-139-5p, miR-181b, miR-181d, miR-199b-5p, miR-200a, miR-448, miR-542-3p, miR-1, miR-137, miR-141, miR-28-5p, miR-370, miR-128, miR-136, miR-148a, miR-181c, miR-185, miR-33b, miR-7, miR-101, miR-148b, miR-152, miR-216b, miR-221, miR-222, miR-33a, miR-431, miR-708, miR-98,
**COL4A1**	69	miR-590-3p, miR-203, miR-410, miR-186, miR-539, miR-340, miR-106a, miR-17, miR-20a, miR-20b, miR-93, miR-106b, miR-23a, miR-23b, miR-29b, miR-374a, miR-519d, miR-9, miR-29a, miR-29c, miR-374b, miR-454, miR-495, miR-544, miR-144, miR-200b, miR-381, miR-429, miR-543, miR-129-5p, miR-1297, miR-494, miR-132, miR-182, miR-26a, miR-26b, miR-300, miR-181b, miR-181d, miR-200c, miR-212, miR-326, miR-330-5p, miR-506, miR-125b, miR-137, miR-21, miR-370, let-7g, let-7i, miR-10a, miR-10b, miR-125a-5p, miR-128, miR-148a, miR-181c, miR-33b, miR-411, miR-421, miR-124, miR-148b, miR-152, miR-16, miR-195, miR-33a, miR-377, miR-424, miR-590-5p, miR-98,
**COL4A3**	69	miR-590-3p, miR-203, miR-410, miR-186, miR-539, miR-340, miR-106a, miR-17, miR-20a, miR-20b, miR-93, miR-106b, miR-29b, miR-374a, miR-519d, miR-9, miR-130a, miR-29a, miR-29c, miR-301a, miR-301b, miR-374b, miR-454, miR-495, miR-544, miR-144, miR-200b, miR-381, miR-429, miR-1297, miR-130b, miR-132, miR-26a, miR-26b, miR-300, miR-135a, miR-135b, miR-139-5p, miR-200a, miR-200c, miR-212, miR-326, miR-330-5p, miR-506, miR-542-3p, miR-1, miR-125b, miR-137, miR-141, miR-146b-5p, miR-154, miR-216a, miR-370, miR-376c, miR-103, miR-107, miR-122, miR-125a-5p, miR-140-5p, miR-143, miR-146a, miR-206, miR-613, miR-653, miR-223, miR-25, miR-299-3p, miR-377, miR-384,
**ANK2**	68	miR-590-3p, miR-203, miR-186, miR-106a, miR-17, miR-20a, miR-20b, miR-93, miR-106b, miR-23a, miR-23b, miR-519d, miR-9, miR-495, miR-544, miR-144, miR-200b, miR-429, miR-129-5p, miR-1297, miR-302c, miR-302d, miR-372, miR-373, miR-182, miR-26a, miR-26b, miR-302a, miR-302b, miR-302e, miR-520a-3p, miR-520b, miR-520c-3p, miR-520d-3p, miR-520e, miR-139-5p, miR-199b-5p, miR-200c, miR-34a, miR-448, miR-125a-3p, miR-146b-5p, miR-28-5p, miR-34c-5p, miR-376c, let-7g, let-7i, miR-122, miR-128, miR-136, miR-140-5p, miR-146a, miR-148a, miR-33b, miR-449a, miR-449b, miR-7, miR-148b, miR-152, miR-16, miR-195, miR-199a-5p, miR-25, miR-27a, miR-27b, miR-33a, miR-424, miR-708,
**WNK3**	52	miR-590-3p, miR-203, miR-186, miR-539, miR-340, miR-106a, miR-17, miR-20a, miR-20b, miR-93, miR-106b, miR-23a, miR-23b, miR-519d, miR-130a, miR-301a, miR-301b, miR-454, miR-144, miR-381, miR-543, miR-1297, miR-130b, miR-494, miR-132, miR-26a, miR-26b, miR-300, miR-139-5p, miR-199b-5p, miR-212, miR-542-3p, miR-1, miR-146b-5p, miR-21, miR-485-5p, let-7g, let-7i, miR-10a, miR-10b, miR-206, miR-411, miR-488, miR-613, miR-101, miR-16, miR-195, miR-199a-5p, miR-211, miR-424, miR-590-5p, miR-98,
**COL3A1**	49	miR-590-3p, miR-203, miR-410, miR-186, miR-340, miR-29b, miR-9, miR-29a, miR-29c, miR-495, miR-200b, miR-381, miR-429, miR-129-5p, miR-300, miR-135a, miR-135b, miR-181b, miR-181d, miR-199b-5p, miR-200c, miR-326, miR-330-5p, miR-1, miR-154, miR-21, miR-28-5p, let-7g, let-7i, miR-103, miR-107, miR-122, miR-128, miR-136, miR-181c, miR-33b, miR-421, miR-488, miR-653, miR-16, miR-195, miR-199a-5p, miR-211, miR-33a, miR-424, miR-431, miR-590-5p, miR-708, miR-98,
**COL4A4**	43	miR-590-3p, miR-203, miR-410, miR-539, miR-23a, miR-23b, miR-29b, miR-374a, miR-130a, miR-29a, miR-29c, miR-301a, miR-544, miR-130b, miR-182, miR-139-5p, miR-181d, miR-34a, miR-448, miR-506, miR-125a-3p, miR-125b, miR-34c-5p, miR-103, miR-107, miR-10a, miR-10b, miR-206, miR-449a, miR-449b, miR-488, miR-653, miR-101, miR-124, miR-195, miR-216b, miR-221, miR-222, miR-25, miR-299-3p, miR-361-5p, miR-384, miR-424,
**SNCA**	42	miR-186, miR-539, miR-340, miR-106a, miR-17, miR-20a, miR-20b, miR-93, miR-106b, miR-23a, miR-23b, miR-29b, miR-374a, miR-519d, miR-9, miR-130a, miR-374b, miR-454, miR-495, miR-144, miR-429, miR-129-5p, miR-494, miR-182, miR-34a, miR-125a-3p, miR-216a, miR-34c-5p, miR-485-5p, miR-148a, miR-449a, miR-449b, miR-488, miR-7, miR-101, miR-148b, miR-152, miR-221, miR-222, miR-223, miR-361-5p, miR-431,
**ATXN1**	39	miR-590-3p, miR-106a, miR-17, miR-20a, miR-20b, miR-93, miR-23a, miR-23b, miR-374a, miR-130a, miR-301a, miR-301b, miR-374b, miR-144, miR-543, miR-130b, miR-302d, miR-373, miR-132, miR-182, miR-181b, miR-181d, miR-200a, miR-326, miR-330-5p, miR-542-3p, miR-141, miR-28-5p, miR-103, miR-107, miR-181c, miR-185, miR-421, miR-653, miR-101, miR-211, miR-221, miR-222, miR-708,
**ACTN2**	39	miR-203, miR-186, miR-539, miR-23a, miR-23b, miR-374a, miR-9, miR-301a, miR-301b, miR-495, miR-200b, miR-429, miR-543, miR-129-5p, miR-1297, miR-302c, miR-302d, miR-372, miR-373, miR-26a, miR-26b, miR-302a, miR-302b, miR-302e, miR-520a-3p, miR-520b, miR-520c-3p, miR-520d-3p, miR-520e, miR-181b, miR-181d, miR-199b-5p, miR-137, miR-370, miR-143, miR-181c, miR-421, miR-199a-5p, miR-384,
**CAV1**	39	miR-203, miR-340, miR-301b, miR-454, miR-544, miR-144, miR-543, miR-302c, miR-302d, miR-372, miR-373, miR-302a, miR-302b, miR-302e, miR-520a-3p, miR-520b, miR-520c-3p, miR-520d-3p, miR-520e, miR-199b-5p, miR-34a, miR-448, miR-506, miR-125a-3p, miR-154, miR-216a, miR-28-5p, miR-485-5p, miR-128, miR-136, miR-411, miR-421, miR-7, miR-124, miR-199a-5p, miR-223, miR-27a, miR-27b, miR-361-5p,
**ATP1A2**	37	miR-203, miR-106a, miR-17, miR-20a, miR-20b, miR-93, miR-106b, miR-519d, miR-130a, miR-301a, miR-301b, miR-454, miR-1297, miR-130b, miR-182, miR-26a, miR-26b, miR-135a, miR-135b, miR-34a, miR-154, miR-34c-5p, miR-370, miR-485-5p, miR-122, miR-148a, miR-411, miR-449a, miR-449b, miR-148b, miR-152, miR-216b, miR-25, miR-27a, miR-27b, miR-299-3p, miR-384,
**COL4A2**	33	miR-186, miR-106a, miR-17, miR-20a, miR-20b, miR-93, miR-106b, miR-23a, miR-23b, miR-29b, miR-519d, miR-9, miR-29a, miR-29c, miR-1297, miR-130b, miR-302c, miR-302d, miR-372, miR-373, miR-26a, miR-26b, miR-302a, miR-302b, miR-302e, miR-520a-3p, miR-520b, miR-520c-3p, miR-520d-3p, miR-520e, miR-216a, miR-140-5p, miR-377,
**CACHD1**	32	miR-590-3p, miR-203, miR-186, miR-144, miR-200b, miR-429, miR-543, miR-494, miR-200a, miR-200c, miR-326, miR-330-5p, miR-34a, miR-542-3p, miR-1, miR-141, miR-34c-5p, miR-370, let-7g, let-7i, miR-136, miR-143, miR-206, miR-33b, miR-411, miR-449a, miR-449b, miR-488, miR-613, miR-27a, miR-27b, miR-33a,
**PDGFB**	30	miR-590-3p, miR-410, miR-186, miR-340, miR-106a, miR-17, miR-20a, miR-20b, miR-93, miR-106b, miR-519d, miR-9, miR-200b, miR-129-5p, miR-1, miR-154, miR-21, miR-34c-5p, let-7g, let-7i, miR-10a, miR-10b, miR-122, miR-140-5p, miR-143, miR-206, miR-488, miR-613, miR-211, miR-98,
**TGFBI**	29	miR-539, miR-106a, miR-17, miR-20a, miR-20b, miR-93, miR-106b, miR-519d, miR-9, miR-544, miR-200b, miR-429, miR-543, miR-494, miR-132, miR-181b, miR-181d, miR-200c, miR-212, miR-34a, miR-542-3p, miR-21, miR-34c-5p, miR-485-5p, miR-181c, miR-449a, miR-449b, miR-222, miR-590-5p,
**VDR**	27	miR-374a, miR-374b, miR-544, miR-129-5p, miR-302c, miR-302d, miR-372, miR-373, miR-494, miR-302a, miR-302b, miR-302e, miR-520a-3p, miR-520b, miR-520c-3p, miR-520d-3p, miR-520e, miR-326, miR-330-5p, miR-448, miR-21, miR-485-5p, miR-10a, miR-10b, miR-136, miR-16, miR-223,
**WFS1**	27	miR-106a, miR-17, miR-20a, miR-20b, miR-93, miR-106b, miR-519d, miR-302c, miR-302d, miR-372, miR-373, miR-302a, miR-302b, miR-302e, miR-520a-3p, miR-520b, miR-520c-3p, miR-520d-3p, miR-520e, miR-506, miR-216a, miR-10a, miR-124, miR-211, miR-361-5p, miR-377, miR-431,
**COL1A1**	23	miR-590-3p, miR-186, miR-29b, miR-9, miR-29a, miR-29c, miR-381, miR-129-5p, miR-494, miR-132, miR-182, miR-300, miR-212, miR-154, miR-216a, miR-376c, let-7g, let-7i, miR-103, miR-107, miR-143, miR-185, miR-98,
**COL6A2**	11	miR-340, miR-29b, miR-29a, miR-29c, miR-132, miR-181b, miR-181d, miR-212, miR-185, miR-7, miR-431,

### Integrated miRNA/mRNA analysis

MiRNAs have been shown to play an important role in regulating the expression of targeted genes. Therefore, it is necessary to search for novel miRNAs associated with the diseases of interest. miRNA and mRNA interaction networks were used to explore the cross-talk mechanism of miRNAs with the twenty hub genes (Figure 5B-a). The network communication was visualized using the R software package visNetwork 2.0 [12], and a sub-network of the top five hub genes with a certain degree of connectivity of 5 was extracted (Figure 5B-b). Next, the top 10 most-expressed hub genes for these data profiles were tested using the two- tailed T-test (*p* <0.05), including ACTN2, ANK2, ATXN1, COL1A1, COL4A4, COL6A2, CXCL12, SNCA, VDR, WFS1 (Figure 5B-c).

These top 10 hub genes were then verified on clinical samples. The Real-time PCR was used to assess the mRNA expression of these genes (Figure 5C). Western Blot analysis was used to evaluate the levels of top 10 most-expressed hub genes in CAVS (Figure 5D), consistent with our data in Figure 5B.

The most relevant miRNAs of these hub genes were detected, including miR-590-3p, miR-374a, miR-374b, miR-340, miR-203, miR-144, miR-494, miR-539, miR-410, and miR-181d (Table 2). Additionally, an integrated heatmap of miRNA/mRNA was generated based on the expression of the hub genes and the miRNA conservation scores (Supplementary Figure 2). Combination of the miRNA conservation scores was used to distinguish which miRNAs can finally be used as important targets.

Taken together, our data showed that the occurrence of mitochondrial dysfunction after CAVS-induced myocardial ischemia is likely associated with these significantly different mitochondrial function-related proteins and the upstream regulatory miRNAs.

## DISCUSSION

In this study, three gene expression profiles of aortic valve tissue in patients with and without CAVS were included. Functional enrichment analysis demonstrated that the mitochondrial function variation plays an important role in the pathogenesis of CAVS. Moreover, the integrated miRNA/mRNA analysis results uncovered several miRNAs and genes that can be used as diagnostic biomarkers or in therapeutic approaches.

**Figure 6:**
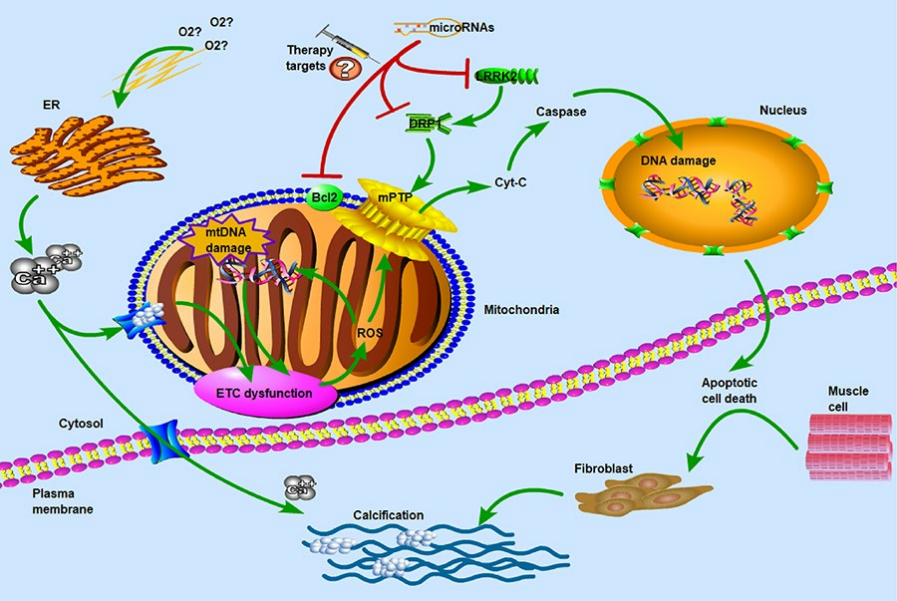
**Molecular and subcellular events leading to apoptosis and calcification.** ETC: electron transport chain; ROS: reactive oxygen species; ER: endoplasmic reticulum; mPTP: mitochondrial permeability transition pore; LRRK2: leucine-rich repeat kinase 2; DRP1: dynamin-related protein-1. Hypoxia and ER stress had been known as the key factors in calcium overflow. ETC dysfunction and ROS reaction induced the opening of mPTP pores to release of Cyt-C which was thought to be important in the apoptotic process. DRP1 and LRRK2 were two major dysregulated genes in CAVS patients and may be used as potential therapeutic targets for microRNAs. The main of our findings have revealed the critical roles of miRNAs in the regulation of target genes that influence the mitochondrial function and muscle cell death. Concomitant with the overflow of calcium ions, the calcification of biological process was induced to activate. Therefore, hypoxia and calcification formed a vicious-loop in cardiovascular disease, exacerbating stenosis and calcification of heart valves.

It has been reported that mitochondrial dynamics plays an important role in cells with high energy demands, such as cardiomyocytes [13,14] and skeletal muscle cells [15,16]. The balance of mitochondrial fission and fusion has been identified as an important mechanism for maintaining normal mitochondrial numbers and morphology, both of which are necessary for maintaining cardiomyocyte integrity [17]. Progressive damage of mitochondrial function is a hallmark of cardiac remodeling and occurs during the entire CAVS pathological process, which includes persistent muscle dysfunction, heart failure and eventually death [18]. Mitochondrial permeability transition pore (mPTP) opening is dependent on elevated Ca^2+^ levels [19] and induces the release of cytochrome C [20], which is considered important for the apoptotic process [21]. Accordingly, this disorganized environment might induce fibroblasts to produce more collagen (Figure 6).

Previous studies showed that collagens are related to Ca^2+^-mediated mitochondria and sarcoplasmic reticulum dysfunctions, the deficiency of autophagy, inappropriate mPTP opening, etc. COL1A1 was reported to be involved in eNOS-related mitochondrial function regulation and the oxidative properties [22,23]. COL6A2 Knock-in revealed abnormal mitochondria in muscle biopsy [24]. Both of these two collagens were also found different expression in integrated miRNA/mRNA analysis, indicating the critical role of mitochondrial functions in CAVS pathological process.

Leucine-rich repeat kinase 2 (LRRK2), one of the hub genes involved in mitochondrial membrane function (Figure 5A), has been reported to play a crucial role in inducing mitochondrial fragmentation by mitochondrial dynamin-related protein-1 (Drp1) in patients with Parkinson’ disease [25,26]. LRRK2 is a large multi-domain protein kinase that contains an Ankyrin (ANK) repeat region [27]. ANK was reported to be related to the localization and membrane stabilization of ion transporters and ion channels in cardiomyocytes. The study of Stephan R et al. [28] showed that ANK2 plays an important role in the dimensions of axons as well as synaptic terminals and lack of ANK2 may limit anterograde transport velocities of mitochondria and synaptic vesicles. Our results also showed a decrease in ANK2 expression in CAVS, suggesting that the mitochondria and vesicle transport may be disrupted in myocardial ischemia induced by CAVS.

It was reported that lack of ataxin-1 protein (ATXN1) induced early alterations in ATP synthesis and oxidative stress, which may be regulated by GSK3β and PI3K/Akt/mTOR pathways [29,30]. The study of Ma J et al. [31] indicated that CXCL12 may polarize mtDNA and modulate mitochondrial respiration [32]. A study [33] also showed that CXCL12 could induce apoptosis of bone marrow stem cells (BMSCs) through mitochondrial pathway (PI3K/Akt and ERK1/2) after hypoxia. The studies mentioned above further confirmed the mitochondrial disorder in myocardial tissues of CAVS and PI3K/Akt/mTOR pathway may influence the process of mitochondrial dysfunctions after myocardial ischemia. As a classical mitochondrial pathway, our previous study showed that the mutation in PI3K (PIK3CA) plays a vital role in several diseases, especially in colorectal cancers [34]. Whether PIK3CA mutation affects myocardial resistibility to ischemia or hypoxia and thereby influences the survival conditions after ischemic injuries need further investigation.

SNCA is detected to be associated with mitophagy [35] and mitochondrial dynamics in several diseases. The study of YX Gui et al. demonstrated that SNCA overexpression not only altered mitochondrial morphology but also significantly increased the translocation of Drp1. The extracellular signal-regulated kinase (ERK) was confirmed to be involved in the regulation of Drp1 and SNCA-mediated neurotoxicity [36]. Actinin Alpha 2 (ACTN2) is a F-actin cross-linking protein which is expressed in myocardium [37] and helps to anchor myofibrillar actin filaments from the adjacent sarcomeres to Z-discs [38]. Recent studies [39] have demonstrated that ACTN2 deficiency affects the binding characteristics of actin as well as cardiomyocyte Z-discs and influences the formation of hypertrophic cardiomyopathy. Our results verified the expression of ACTN2 was suppressed in CAVS patient, which is consistent with the results of previous studies [40]. Yao T et al. [41] has reported the important role of vitamin D receptor (VDR) in ischemia/reperfusion-induced myocardial injury. The endogenous VDR expression was detected in the mouse heart and myocardial ischemia upregulated VDR expression. The pathways that were influenced significantly by WFS1 were related to mitochondrial damage and mitochondrial dynamics [42]. WFS1 deficiency may lead to dramatic changes in mitochondrial dynamics (inhibit mitochondrial fusion, alter mitochondrial trafficking, and augment mitophagy) and induce endoplasmic reticulum (ER) stress [43]. This information suggested that the hub genes in CAVS may be related to mitochondrial dynamics, especially the Drp1 translocation process. But the reason why Drp1 was not included in the top ten hub genes related to myocardial ischemia after CAVS needs further investigation.

Although several miRNAs were found to be beneficial for cardiac regeneration, miRNAs are seldom used in clinical screening [44]. In 2012, Dr. Mauro Giacca laboratory showed that human miRNAs can induce neonatal cardiomyocyte proliferation [45] and proved the ability of miR-590-3p in promoting cardiomyocytes cell cycle re-entry *in vivo*. miR-590-3p has also been reported to contribute to the process of myocarditis and heart dysfunction [46]. The study of Wang J et al. demonstrated that up-regulation of miR-590-3p could increase the expression of peroxisome proliferator-activated receptor γ coactivator-1α (PGC-1α) and the downstream targets of PGC-1α, including nuclear respiratory factor 1 (NRF-1) and mitochondrial transcription factor A (TFAM), which are the key genes regulating mitochondrial function [47]. Our cross-linked miRNAs result in Table 2 demonstrated that miR-590-3p may cross-link with many hub genes in CAVS, such as CXCL12, COL4A4, ATXN1 and COL1A1. Our PCR and WB results also detected the increased expression of these genes in CAVS, indicating that miR-590-3p may be down-regulated in myocardial ischemia after CAVS. Whether the expression of PGC-1α is also down-regulated in the process of mitochondrial dysfunctions after CAVS-induced myocardial ischemia is still unknown.

miRNA-199b-5p is a direct regulator of calcineurin /NFAT and is up-regulated in cardiac tissues of heart failure patients and in animal models of cardiac hypertrophy [48]. Our hub genes and cross-linked miRNAs results in Table 2 also demonstrated that miRNA-199b-5p may directly target ACTN2 and ANK2 genes which are both suppressed in CAVS, suggesting the effects of these molecules on the pathogenesis of aortic stenosis. In addition, miRNA-374b [49], miRNA-340 [50] and miRNA-203 [51] have also been reported to be abnormally expressed in aortic stenosis patients, which can be used as a diagnostic/ prognostic biomarker of CAVS in the future. However, during the process of the disease, genetic alteration may occur secondary to some pathological changes. This notion should be kept in mind during our in-depth dissection of the data. On the other hand, genetic biomarkers of patients prior to AS development appear to be quite useful but it may have no advantage to established imaging examination methods in AS patients who already develop prominent symptoms.

In conclusion, this chip-based analysis identified 471 shared DEGs, whose functional enrichment mainly involve collagen formation and mitochondrial functions. Several novel genes associated with collagen organization and calcium ion transport, such as ANK2 and LRRK2, have been revealed in CAVS. Moreover, miRNA-mRNA cross-linked network analysis also identifies several new miRNAs, including miR-590-3p, miR-374a, miR-374b, miR-340, miR-203, miR-144, miR-494, miR-539, miR-410, and miR-181d, etc., which may play critical roles in the regulation of top ten target hub genes in CAVS (ACTN2, ANK2, ATXN1, COL1A1, COL4A4, COL6A2, CXCL12, SNCA, VDR, WFS1). However, since this study was unable to mimic the CAVS process at animal levels, further confirmation of the results was limited. Despite the fact, our findings may have implications for the diagnosis/prognosis and treatment of CAVS and the mitochondrial function-related proteins such as LRRK2 and ANK2 screened by CAVS bio-signal are important for the regulation of mitochondrial function in CAVS-induced myocardial ischemia.

## MATERIALS AND METHODS

### Materials

Antibodies for ACTN2, ANK2, ATXN1, COL1A1, COL4A4, COL6A2, CXCL12, SNCA, VDR, WFS1 were purchased from Abcam (Cambridge, MA, USA). The genotyping primers of these genes were listed in Supplementary Table 1. GAPDH was used as interior references for myocardial tissues, which were also purchased from Abcam (Cambridge, MA, USA). The ROS fluorescent probes and JC-1 fluorescent probes were purchased from Beyotime Biotechnology (Shanghai, CHINA). MitoTracker Deep Red was purchased from Invitrogen (Carlsbad, CA, USA). The TUNEL detection kit was purchased from Roche (Indianapolis, IN, USA). All other chemicals were purchased from Sigma unless specifically mentioned otherwise.

### Clinical samples

Calcified valve specimens were obtained from patients with CAVS undergoing surgical treatment at the Xinqiao Hospitals of Army Medical University (Chongqing, China). Samples for Non-CAVS group were valves from aortic insufficiency patients, which have no calcification. The study was approved by the Ethics Committee of Institute of Biomedicine Research of Xinqiao Hospitals of the Army Medical University (Chongqing, China). Quantitative real-time polymerase chain reaction (qRT-PCR) and Western blots were performed to verify mRNA and protein expression levels in CAVS and non-CAVS tissues. The nearby myocardial tissues in samples were obtained for electronic microscopy observation.

### Hypoxia treatment

To simulate myocardial ischemia induced by CAVS *in vitro*, we carried out hypoxia treatment. H9C2 were put into a hypoxia compartment, bubbled with hypoxic gas (95% N2—5% CO2) for 15 min and then occluded for 10 min. This procedure was repeated five times until the O2 concentration in the compartment was below 0.2%. After all the procedures, the cells were cultured in this creating hypoxia conditions for 4h [52].

### Determination of mitochondrial transmembrane potential (ΔΨm)

H9C2s were seeded in confocal culture plates at a density of 5×10^4^ cells per well. After hypoxia treatment, the cells were stained with JC-1 (Sigma-Aldrich, St. Louis, MO, USA), a cationic fluorescent dye, and incubated for 20 minutes at 37°C. JC-1 monomer was excited by 488 nm laser and emissions collected at >530 nm. Imaging of JC-1 aggregate was set at 633 nm excitation and 590 nm emission [53].

### Detection of intracellular ROS

The fluorescent probe DCFH-DA (2,7-dichlorofluorescein diacetate, Jiancheng, Nanjing, CHINA) was used to detect intracellular ROS levels according to the protocol provided by the company. DCFH-DA can independently pass through the cell membrane and can be deacetylated to DCFH by esterase. Once deacetylated, DCFH cannot cross the cell membrane again and becomes deposited in the cells. Because ROS can oxidize non-fluorescent DCFH to fluorescent DCF, the fluorescence intensity is correlated with the intracellular ROS level. Briefly, 5×10^4^ cells were placed on confocal culture plate overnight in the incubator at 37°C and DCFH-DA was dissolved in DMSO at a concentration of 20 mM as a stock solution. Then, the culture plate with cells was suspended in 1 ml PBS with 20uM of DCFH-DA and incubated for 30 min at 37 °C. Samples were washed with 1ml PBS 3 times and fluorescence microscope using 488 nm laser for excitation and 510-550 nm for emission [54].

### Detection of mPTP opening

mPTP opening detection was based on the protocol previously [55]. Hypoxia-treated H9C2s were co-incubated with Calcein (2μM) and MitoTracker (100nM) for 30 minutes. After washing twice with PBS, the cells were then exposed to CoCl2 (2mM) for 15min to detect the distribution of cobalt inside mitochondria. The mPTP opening degrees were reflected by Red (MitoTracker)/ Green (Calcein) fluorescence.

### TUNEL staining

H9C2s were incubated on petri dishes and fixed with 4% paraformaldehyde at room temperature for 60min. After washing 3 times with PBS for 5min, cells were incubated with 0.1% Triton-100 PBS for 5min in ice bath. The TUNEL detection solutions were prepared based on the instructions (in situ cell death detection kit, Roche, Indianapolis, IN, USA) and added 50ul into each petri dish. Wash 5min for 3 times after incubation and add DAPI to stain nuclei. The TUNEL florescent probe was excited by 488 nm laser and the detection wavelength was set from 515 to 565nm.

### Data expression profiles selection

We queried the database of Gene Expression Omnibus (GEO) using the keywords “aortic stenosis” and “calcified” to screen for related data profiles. Three GEO data profiles (GSE12644 [56], GSE51472 [57], and GSE83453 [58]) (Table 1) including four time-stamp datasets were associated with CAVS. Forty-seven men aged 46-73 from Canada and Finland were allocated to four paired groups. Due to the two different platforms used, these gene expression profiles were normalized to same levels and analyzed using edgeR [59] package of R software (R version 3.4.3 -- "Kite-Eating Tree").

### Differentially expressed genes (DEGs) in calcified aortic valve samples

Gene expression matrices of CAVS samples were investigated and shared DEGs were screened with the same criteria (*p* < 0.05) after quality filtering and normalization through robust multi-array averaging. The common DEGs in the four paired groups were mainly used to explore the hub genes and their highly-cross-linked miRNAs. Subsequently, gene annotation and functional enrichment analysis of the biological process, molecular function, and cellular composition of these hub genes was performed based on all background genes. In addition, a subset of shared DEGs associated with CAVS was used to integrate the miRNA/mRNA analysis and to develop novel target miRNAs and genes.

### Construction of co-expression networks

We calculated paired genetic correlations based on WGCNA (Weighted Gene Co-expression Network Analysis) [60] to reveal the Pearson correlation co-efficient between gene expression profiles [61]. The power β =10 was selected to generate a scale-free topology overlap matrix (TOM) and average linkage hierarchical clustering, a reliable measure of network interconnectivity [62], was used to detect gene modules representing the topological overlap between shared genes. Gene modules were displayed as branches of a dendrogram, which was produced by Dynamic Tree-Cut algorithm [63]. To characterize each gene module, the significance and correlation of module eigengenes were generated, which is considered as the first major component of a given module and is a characteristic representation of the gene expression profile that captures significant changes in the module.

### Functional investigation of hub genes

Hub genes are genes that are highly connected with many other adjacent genes in a co-expressed network, which show significant association with module eigengenes and have high intramolecular connectivity. After screening for the common genes of the included data profiles, a gene co-expression network analysis was conducted to reveal the hub genes of these DEGs. We also performed a functional enrichment analysis of shared DEGs with *p*<0.05 using clusterProfiler software package [64] This functional enrichment analysis aims to reveal significant gene terminologies and to discover important biological pathways and molecular functions associated with CAVS.

### Integrated miRNA/mRNA analysis

MRNA and miRNA cross-talk have been used as a novel method to explore new biomarkers in relation to the biological processes based on Internet sources, such as miRanda and miRwalk. The miRanda database (targets and expression) has been used to search for the interplay between mRNAs and miRNAs, and such research would contribute to finding novel regulatory interactions between them.

### Statistics analysis

Results were expressed as means and standard deviations (SD). One-way analysis of variance (ANOVA) was used for experiments with more than two groups and followed by Tukey’s post hoc analysis. The survival analysis was calculated by Kaplan-Meier method using SPSS (SPSS Inc., Chicago, IL, USA). *p*<0.05 was considered statistically significant.

## SUPPLEMENTARY MATERIAL

Supplementary File
