# Supplementary material for: miRNA-mRNA crosstalk in myocardial ischemia induced by calcified aortic valve stenosis
Source: Aging (Albany NY). 2019 Jan 16;11(2):448–66. doi: 10.18632/aging.101751 (PMC6366972; doi:10.18632/aging.101751)
Supplement: Supplementary File [file aging-11-101751-s001.pdf]

SUPPLEMENTARY MATERIAL

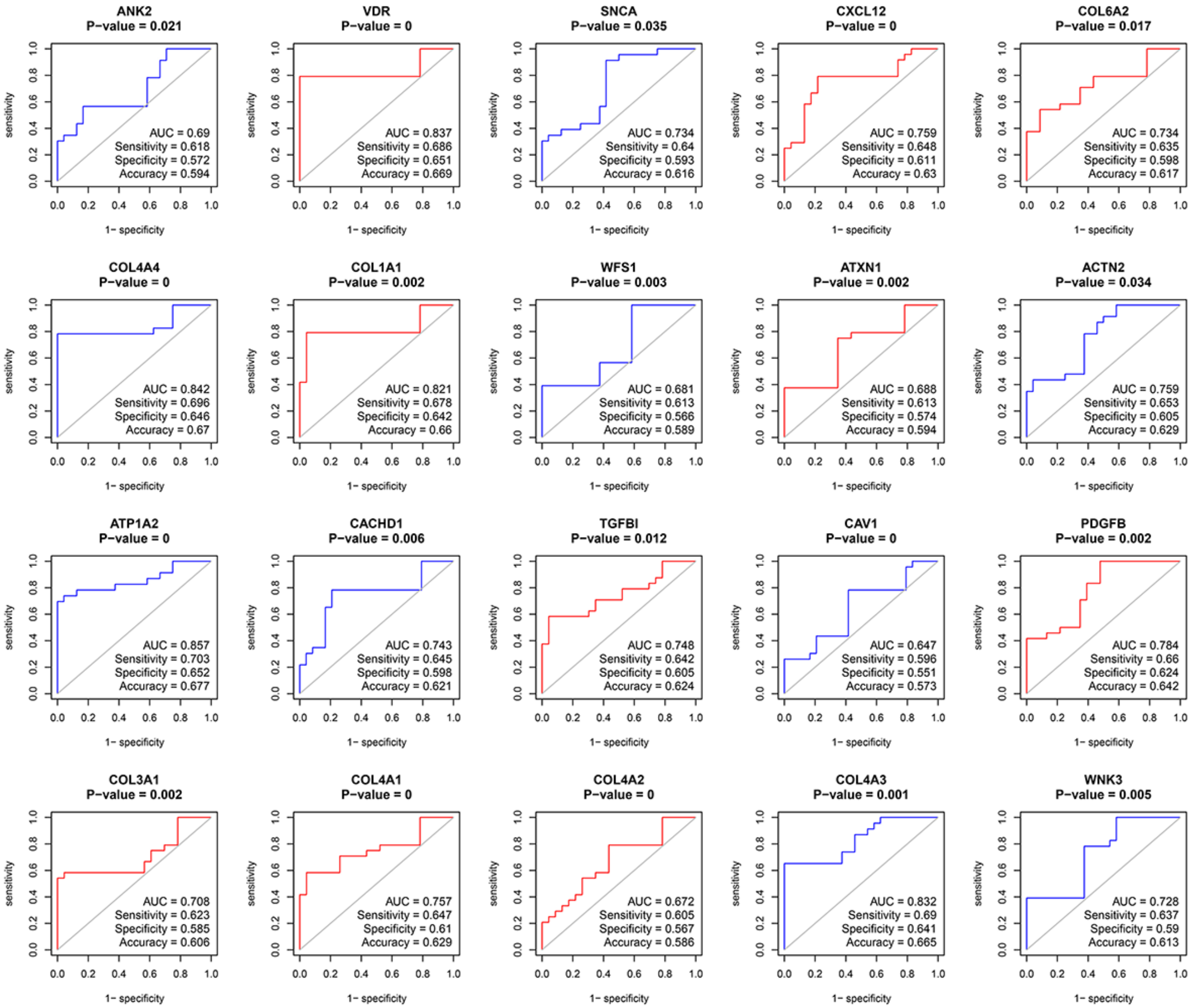

Supplementary Figure 1. Diagnostic characteristics of the twenty hub genes. Diagnostic characteristics including sensitivity, specificity, accuracy and area under curve (AUC). Red and blue curves indicate up-regulated and down-regulated DEGs, respectively.

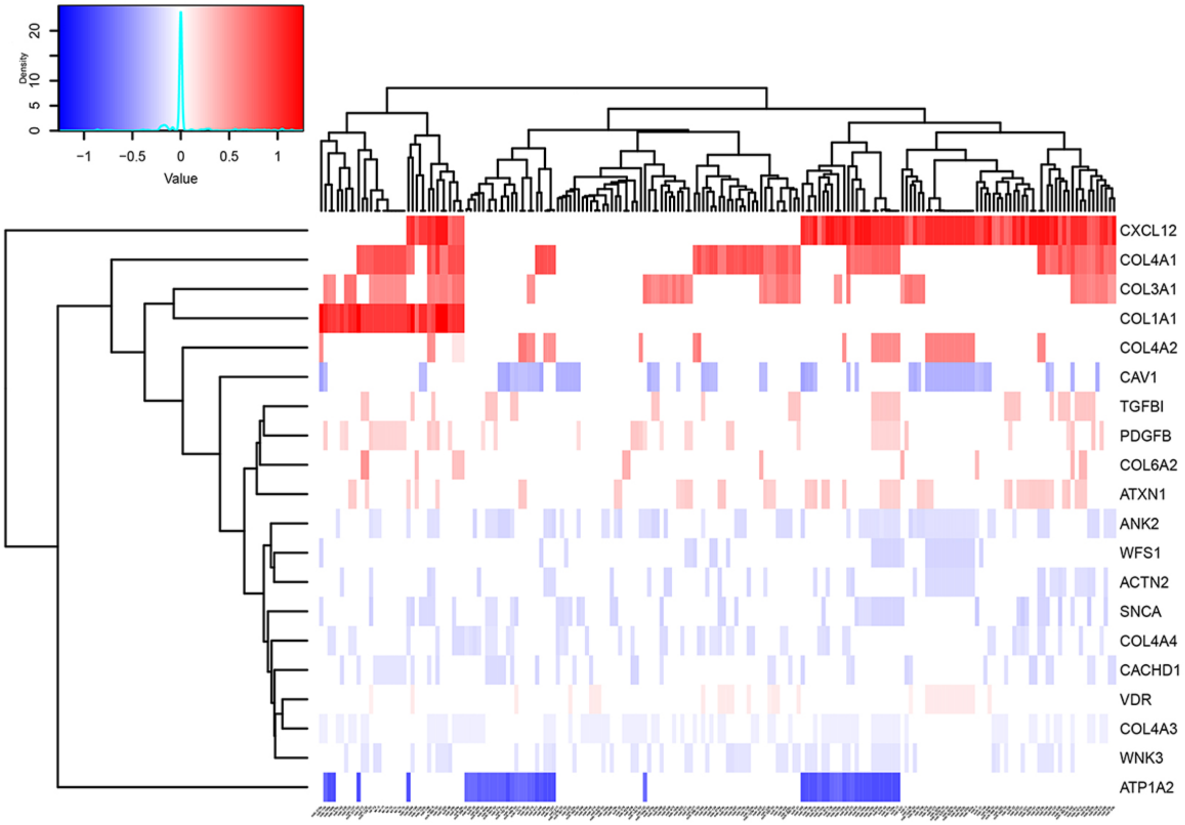

Supplementary Figure 2. Correlation of hub genes and highly connected miRNAs. Each of the hub gene expression level is revaluated with its corresponding miRNA and represented by the color's saturation. The relative changed levels represented by the legend's color saturation.

Supplementary Table 1. The genotyping primer lists of the top ten hug genes.

Genes	genotyping primers	Genes	genotyping primers
ACTIN2	F: CAA ACT CCG TCA CCC TCA GT	COL6A2	F: AGC TCC GTG CTC CTG CTC TG
	R: GGT GCC AGT GGT TTC TTG TT		R: ATG GTG ACG CTC TCC GAG GTG
ANK2	F: AAG GCA CCT GTT GAT GAT GTC ACC	CXCL12	F: TCG TGG TCG TGG TCC TC
	R: CTG GCG TTC GGA TTG GCT CTC		R: TTG AGA TGC TTG ACG TTG GCT CTG
ATXN1	F: GTC GGC AGC ACT GAC ATG GAA G	SNCA	F: GTG GCA ACA GTG GCT GAG AAG AC
	R: CGG TGG TGA AGA ACG TGT GAG		R: CCA GTG GCT GAG AAG AC
COL1A1	F: GCG AGA GCA TGA CCG ATG GAT TC	VDR	F: CAA GGA CAA CCG ACG CCA CTG
	R: GCC TTC TTG AGG TTG CCA GTC TG		R: CCT CCT CCT CCT TCC GCT TCA G
COL4A4	F: GGT GAG GTT GGT CAG CAA GGT TC	WFS1	F: CAA GGT GTT CCA GGA CAG CAA GG
	R: CCT GGT GGT CCT GGT AGT CC		R: GGT GGT TCC AGC CGA AGT TGA C
